# A Novel Methodology for Measuring Ambient Thermal Effects on Machine Tools †

**DOI:** 10.3390/s24072380

**Published:** 2024-04-08

**Authors:** Fernando Egaña, Unai Mutilba, José A. Yagüe-Fabra, Eneko Gomez-Acedo

**Affiliations:** 1 Department of Mechanical Engineering, Tekniker, Basque Research and Technology Alliance (BRTA), C/Iñaki Goenaga 5, 20600 Eibar, Spain; unai.mutilba@tekniker.es (U.M.); eneko.gomez-acedo@tekniker.es (E.G.-A.); 2 I3A, Universidad de Zaragoza, C/María de Luna 3, 50018 Zaragoza, Spain; jyague@unizar.es

**Keywords:** machine tool accuracy, thermal error analysis, MIIM methodology

## Abstract

Large machine tools are critically affected by ambient temperature fluctuations, impacting their performance and the quality of machined products. Addressing the challenge of accurately measuring thermal effects on machine structures, this study introduces the Machine Tool Integrated Inverse Multilateration method. This method offers a precise approach for assessing geometric error parameters throughout a machine’s working volume, featuring a low level of uncertainty and high speed suitable for effective temperature change monitoring. A significant innovation is found in the capability to automatically realise the volumetric error characterisation of medium- to large-sized machine tools at intervals of 40–60 min with a measurement uncertainty of 10 µm. This enables the detailed study of thermal errors which are generated due to variations in ambient temperature over extended periods. To validate the method, an extensive experimental campaign was conducted on a ZAYER Arion G™ large machine tool using a LEICA AT960™ laser tracker with four wide-angle retro-reflectors under natural workshop conditions. This research identified two key thermal scenarios, quasi-stationary and changing environments, providing valuable insights into how temperature variations influence machine behaviour. This novel method facilitates the optimization of machine tool operations and the improvement of product quality in industrial environments, marking a significant advancement in manufacturing metrology.

## 1. Introduction

Thermo-elastic deformation, arising from fluctuating ambient conditions prevalent on the majority of large-scale shop floors, poses a significant limitation to achieving manufacturing precision [1]. As stated by Mayr et al. [2], variations in workshop temperatures, coupled with heat generated during metal-cutting operations, can contribute up to 75% of the total geometric inaccuracies in machine tools (MTs). An increasing demand for the manufacture of large and highly precise components in strategic sectors like energy and aeronautics or major scientific facilities [3,4] necessitates the use of larger MTs and workshops. This amplifies potential error sources, notably those related to ambient temperature variations. In many cases, workshops lack thermal control due to the substantial initial investment and ongoing operational costs required [5,6]; therefore, responsibility lies with the manufacturer to ensure the reliable operation of the MT amidst environmental thermal fluctuations. Enhancing the accuracy of MTs is crucial for both manufacturers and end users to ensure that manufactured products meet specifications.

While the impact of environmental temperature variations on MT performance is a recognised issue, most prior research has primarily concentrated on internal heat sources during the metal-cutting process rather than examining the effects of external temperature fluctuations. This research gap concerning environmental temperature variation and its influence on MT performance, as identified by Gross et al. [1], has led to an incomplete understanding of how to mitigate these effects and enhance the precision of MTs.

A recent comprehensive review by W. Gao et al. [7] discusses advanced technologies for the calibration of machine tools, encompassing thermal errors from various perspectives. This review recognises that although it was introduced in the 1960s by J. Bryan [8], the topic remains an actively researched area to date. Moreover, Mayr et al. [2] emphasise the scarcity of measurement methods capable of accurately assessing thermal errors induced by these variations. They also emphasised the necessity of a measurement that can capture all pertinent geometrical error parameters, encompass an entire working volume, maintain low measurement uncertainty, and deliver fast results to monitor the impact of temperature changes effectively.

ISO Standard 230-3:2020 [9] outlines a method of assessing thermal distortion in MTs caused by environmental temperature variations. However, it only evaluates errors in five degrees of freedom at a single point between the tool and the workpiece. This approach is suitable for small-sized MTs but lacks insight into the “relevant working volume”, rendering it insufficient for medium and large MTs. In line with this standard measurement approach, Wei et al. [10,11] recently introduced a notable research study that models and compensates for thermal errors in machine tool spindles, particularly in the presence of strong seasonal variations in ambient temperature, introducing the concept of Ambient Temperature Intervals (ATIs). Their work involved conducting year-round experiments to develop a robust model and compare it with other state-of-the-art algorithms, including deep-learning methods, thus demonstrating the effectiveness of their proposal. 

In recent years, a multilateration measurement scheme employing tracking interferometers (TIs) [12] emerged as a promising solution for characterising and calibrating large MTs. This method measures the length between a retro-reflector, ideally situated at the tool centre point (TCP) on an MT holder, and at least one fixed TI on the base while moving the machine to predetermined points across the relevant working volume. If the measurement procedure is conducted sequentially, it requires at least four repetitions which involve relocating the TI to different positions on the MT. This approach is quite time-consuming, reducing its suitability for assessing thermally induced volumetric errors, as mentioned by W. Gao et al. [7]. Gross et al. [1] examined the impact of ambient temperature variations on MT accuracy by subjecting an MT to various constant temperatures within a climate simulation chamber. They utilised a sequential multilateration scheme employing a Laser Tracer™ for their investigation. If four TIs are accessible, conducting the measurement procedures simultaneously facilitates the swift characterisation of the MT, reducing the overall time required to under 40 min (subject to the MT’s size and the number of measurable points), thereby minimising the influence of ambient temperature variations. 

Schwenke et al. proposed the use of an absolute interferometry-based measurement technology called the Multiline™ system to measure the thermal distortion of MT structures by employing multiple measurement lines attached to the main components of the MT structure [13,14]. Ibaraki et al. proposed a rapid test to explore the impact of spindle heat on an MT’s trajectory on a 2D plane using only one TI [15]. The uncertainty of this method was enhanced by Mori et al. [16] through an additional displacement measurement, rendering it applicable to MTs without rotary tables, though it is still only valid for 2D trajectories. Gillory et al. [17] introduced a highly promising absolute multilateration-based coordinate measurement system utilising a single ADM and four measuring heads, thereby mitigating the expense associated with the four TIs required for simultaneous multilateration. Brosed et al. [18] suggested an alternative approach not based on TIs, employing a telescopic instrument for simultaneous laser multilateration. 

Brecher et al. [3] present an interesting machine-integrated direct method [19] considered cost-efficient by the authors and based on a laser source combined with a pentaprism located on a machine table and two position sensor devices (PSDs) mounted on the MT spindle. However, this method is only suitable for small- and medium-sized machines. Another approach demonstrated by Iñigo et al. [20] is based on allowing for the rapid characterisation of thermal error variation over extended periods for a 1 m^3^ volume.

This research experimentally assesses the Machine Tool Integrated Inverse Multilateration (MIIM) method, presented by Mutilba et al. in [21,22], for the characterisation of environmental thermal errors in a relevant working volume. This study was conducted on an industrial shop floor without artificially altering the workshop environment, and the research targeted medium- and large-sized machine tools. By integrating an absolute distance measurement device into the MT spindle, the MIIM method enables the realisation of an integrated MT multilateration scheme. This innovation addresses the limitations of other approaches, such as the use of climate chambers, four simultaneously high-cost TIs, the Multiline™ system, or artifacts which are not suitable for large machines.

Section 2 outlines the test environment and conditions, providing a characterisation of the medium-sized ZAYER ARION G™ MT utilising a LEICA AT960™ laser tracker along with four wide-angle retro-reflectors. Additionally, this section provides a concise description of the methodology employed, specifically the MIIM method.

Section 3 presents results obtained from various scenarios observed during the research. Some scenarios approached a steady condition, aligning with different temperature values previously obtained by Gross et al. [1]. In contrast, other instances reflected non-steady conditions characterised by day-and-night thermal cycles over several days. These observations facilitated an analysis of how MT errors fluctuate in response to variations in environmental temperature.

Section 4 summarises the primary conclusions in detail, emphasising the utility of the MIIM method for characterising thermal errors in large MTs. This section provides a comprehensive overview highlighting the significance and effectiveness of the MIIM approach in assessing thermal errors within substantial MT systems.

## 2. Materials and Methods

The objective of this study is to conduct an empirical assessment of the MIIM method’s efficacy in quantifying ambient thermal effects across large MTs situated in environments that are both representative of and relevant to the industry. This study intentionally avoids the use of additional high-cost resources, such as heating or cooling systems, to artificially create controlled changes in ambient temperature within the large workshop spaces in which the MTs are located. This decision not only significantly reduces testing costs but is also driven by an intent to authenticate the MIIM method’s applicability under typical conditions encountered in industrial settings.

Industrial environments often have heating systems in place to maintain human comfort during colder months but may not be equipped with cooling systems to mitigate higher temperatures. This research, therefore, aims to demonstrate the MIIM method’s practicality and effectiveness in real-world industrial conditions, which are marked by naturally occurring temperature fluctuations.

This study provides a detailed exposition of the measurement methodology, detailing the materials used and the conditions under which tests were conducted. This thorough description ensures a clear understanding of the experimental setup and facilitates the replication of the study, contributing valuable insights into the MIIM method’s utility in accurately measuring ambient thermal effects within industrial MTs experiencing naturally fluctuating temperatures.

### 2.1. Test Conditions—Environment

An experimental study was performed utilising a ZAYER ARION-G™ portal-type 5-axis multitasking MT housed within the facilities of TEKNIKER, a research entity located in northern Spain. This region experiences notable ambient temperature swings from winter to summer, making it an ideal setting for evaluating MT performance under varying thermal conditions. To capture the natural environmental fluctuations, experiments were conducted across different seasons of the year, relying on meteorological temperature forecasts to select suitable test days, rather than artificially manipulating the workshop’s climate.

The MT is positioned in a dedicated workshop designed explicitly for the assembly and testing of large-scale mechatronic systems. This expansive space measures 90 m in length, 14 m in width, and 12.2 m in height, and is outfitted with ground anchors to firmly secure the mechatronic systems being tested. This setup ensures a controlled testing environment, free from the heat flux influences of other production machinery or processes. The thermal conditions within the workshop are primarily influenced by the indoor environment, especially during the summer months. For winter conditions, while a heating system is utilised to ensure the comfort of the personnel, the absence of an air conditioning system means the ambient temperature is not actively regulated to maintain the optimal operational range (typically 20 °C ± 1 °C) during warmer periods.

**Figure 1:**
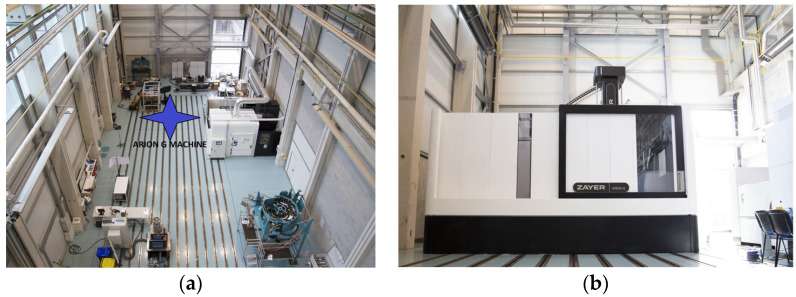
(**a**) Workshop before MT installation and (**b**) MT under study in workshop.

Figure 1 depicts the workshop (a) before the MT installation and (b) the MT installation on the left corner of it.

### 2.2. Research Materials—Machine Tool

The machine tool under investigation, a ZAYER ARION G™, is a 5-axis multitasking portal-type MT that features two main substructures for enhanced functionality and precision. The tool side of the MT incorporates a robust framework consisting of two columns, a crossbeam, a saddle (or Y-slide), and a ram, all anchored firmly to the floor. Conversely, the workpiece side is equipped with a bed, an X-slide, and a rotary table, facilitating a wide range of machining operations.

The operational capacity of the MT is defined by its substantial working volume, which extends to 3.500 mm along the X-axis, 3.100 mm along the Y-axis, and 1.100 mm along the Z-axis. This spacious working area, encompassing the machining spindle, the turning table, and a segment of the ram, is completely closed during operation. This enclosure is designed with the primary goal of safeguarding the health and safety of operators, providing a protective barrier against potential hazards associated with the machining process. However, it is important to note that this enclosure does not incorporate features for thermal control or isolation, focusing instead on ensuring a safe working environment without specifically addressing temperature regulation in its design criteria.

**Figure 2:**
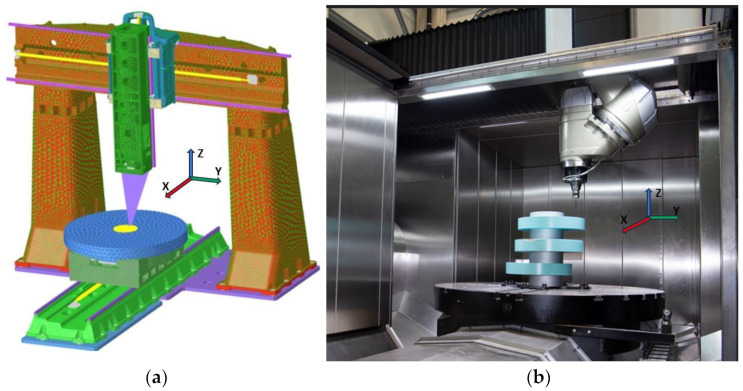
The MT under study: (**a**) a finite element model of the Zayer Arion G™ MT and (**b**) a close view of the MT’s working area with the upper part of the enclosure open [23].

Figure 2 shows the MT under study, providing a finite element model (FEM) of the MT as well as its operational area with the protection enclosure opened.

For a thermal error analysis of the MT, an array of temperature sensors was strategically deployed to meticulously monitor both the structural integrity of the MT and its critical components prone to heat generation. In addition to the existing sensors on the machine, six additional sensors were integrated alongside the machine’s pre-existing sensor suite. Specifically, two of these sensors were dedicated to monitoring ambient temperature: one measured the air temperature inside the working enclosure, and the other assessed the external air temperature outside it.

Table 1 presents the main characteristics of the additional sensors used to complete the thermal characterisation of the machine.

The remaining four sensors were strategically placed on the MT’s substructures to capture precise thermal data from areas of potential heat accumulation. Complementing this setup, the MT’s manufacturer embedded 14 internal temperature sensors within the machine. These sensors play a pivotal role in the machine’s operational safety as they are located in components known for heat production, including the spindle and table bearings, feed drive motors, and ballscrew nuts.

The integration of these sensors provides a comprehensive thermal profile by recording each temperature reading with a configurable timestamp, providing one datum every minute by default. This meticulous data collection enables a detailed analysis, allowing for an exact correlation between temperature fluctuations and the MT’s TCP displacement. Such precision in tracking the temperature/displacement relationship is invaluable in understanding and mitigating thermal errors, ensuring the MT’s optimal performance.

**Figure 3:**
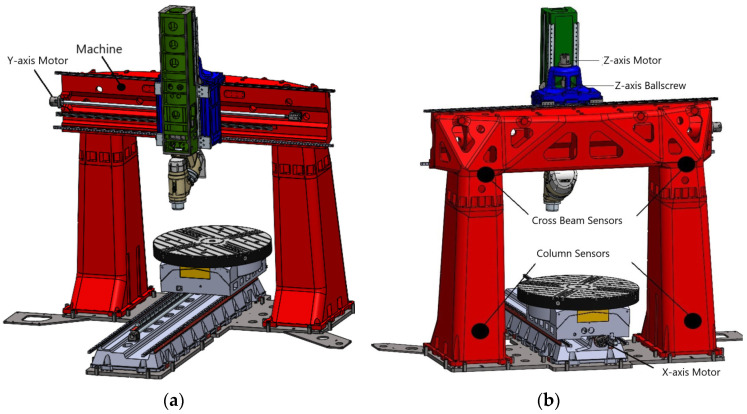
Temperature sensors: (**a**) frontal view of machine; (**b**) back view of machine.

Figure 3 presents a detailed 3D model of the machine, highlighting the integration of key internal temperature sensors as installed by the manufacturer. Additionally, it showcases the strategic placement of four external temperature sensors embedded within the machine’s structure. These supplementary sensors significantly enhance the collection of thermal data throughout the measurement process, enriching the thermal information gathered during the measurements.

**Figure 4:**
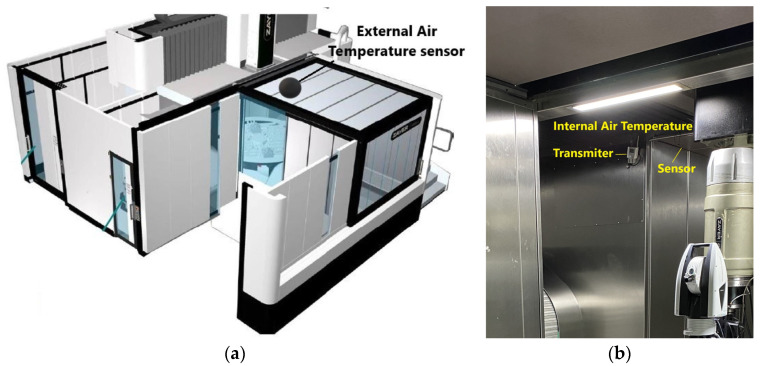
Locations of ambient temperature sensors: (**a**) location of sensor that measures ambient temperature outside working enclosure; (**b**) internal air temperature sensors and wireless transmitter located in working area.

Figure 4 displays the locations of two additional sensors deployed to monitor ambient temperature. One sensor is strategically placed outside the machine’s enclosure, close to it, to capture external ambient conditions. The other sensor measures the air temperature within the enclosure, specifically in the working area.

### 2.3. The Method—MIIM

The Machine Tool Integrated Multilateration (MIIM) method is fundamentally grounded in the multilateration technique, a well-established mathematical approach that utilises pure distance measurements to determine the three-dimensional coordinates of individual points within a measurement space. This methodology shares conceptual similarities with the Global Positioning System (GPS), which employs distance measurements to establish the geographical coordinates of a system on Earth.

Within the specific domain of error mapping in MT through multilateration-based measurements, the integration of a minimum of four strategically positioned fixed points is indispensable. These fixed points serve as reference benchmarks for displacement measurements, which can manifest as either absolute or relative values.

To date, TIs are strategically positioned on the MT table at specified fixed points, and a measuring reflector is attached to the moving spindle to realise mobile points. Typically, only one tracking interferometer is available, necessitating a sequential scheme for multilateration measurements in practical scenarios. However, this sequential approach imposes challenges related to MT repeatability during the data acquisition process, influenced by factors such as thermal drift, mechanical repeatability of the MT, and instability of the measurement system over time.

In contrast, a simultaneous multilateration approach becomes feasible with the provision of four measurement instruments, resulting in a substantial reduction in measurement time to approximately 25% of the total time consumed. In this scenario, the MT volume is traversed once as the four available TIs simultaneously measure the distances from fixed points to the movable reflector. However, a noteworthy constraint of this strategy is associated with the economic implications of acquiring and maintaining four measurement instruments.

The MIIM method, proposed by Mutilba et al. in [21], was developed to overcome inherent limitations in traditional multilateration schemes. These constraints are primarily associated with the manual placement of tracking interferometers on the MT table and the use of a sequential measurement process, both of which contribute to prolonged measurement times and heightened thermal distortion within the MT.

The MIIM methodology represents a paradigm shift by incorporating an absolute interferometer directly into the MT spindle and methodically establishing fiducial points on the machine through the use of wide-angle retro-reflectors. Within this innovative measuring framework, a volumetric point grid designated for measurement comprises locations to which the tracking interferometer is systematically directed. This sequential movement allows the interferometer to obtain precise measurements of the distances to the four fixed fiducial reflectors strategically positioned around the manufacturing system.

The MIIM approach involves integrating an absolute interferometer into the MT spindle and defining several fiducial points on the MT using wide-angle retro-reflectors. This results in a fully autonomous measurement process, reducing the measurement time to a single pass over the MT working volume, taking less than 40 min (depending on the size of the MT and the number of measurement points).

**Figure 5:**
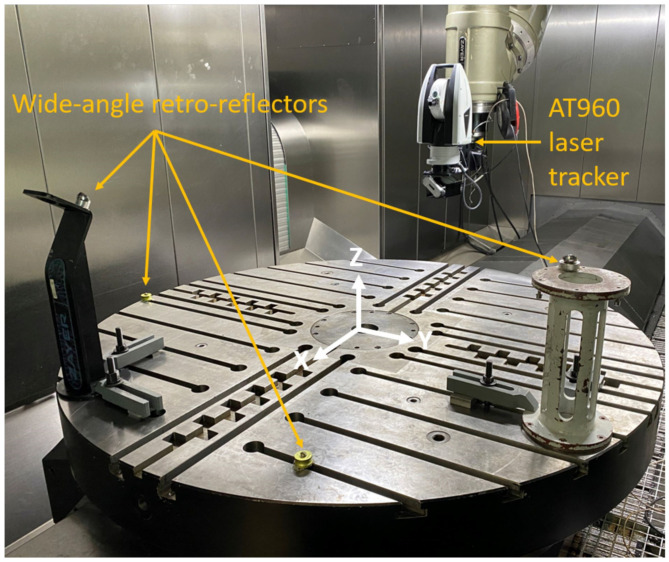
MIIM experimental realisation in ZAYER ARION G™ MT [25].

Figure 5 shows a measurement scenario with the realisation of the MIIM in a ZAYER ARION G large MT with a LEICA AT960™ laser tracker and four wide-angle retro-reflectors located on the machine’s rotating table.

Table 2 presents the main characteristics of the measurement equipment employed during the MIIM tests to measure machine deformations.

The total number of points within the point cloud can be adjusted, and how it is traversed is adaptable according to the specific objective of the test. In scenarios in which temperature changes occur rapidly, fewer points are utilised to expedite measurements and to reduce the overall measurement time of each measurement sequence. In situations involving slower temperature changes and a need for enhanced spatial resolution, a higher number of points in each measurement sequence is needed. 

**Figure 6:**
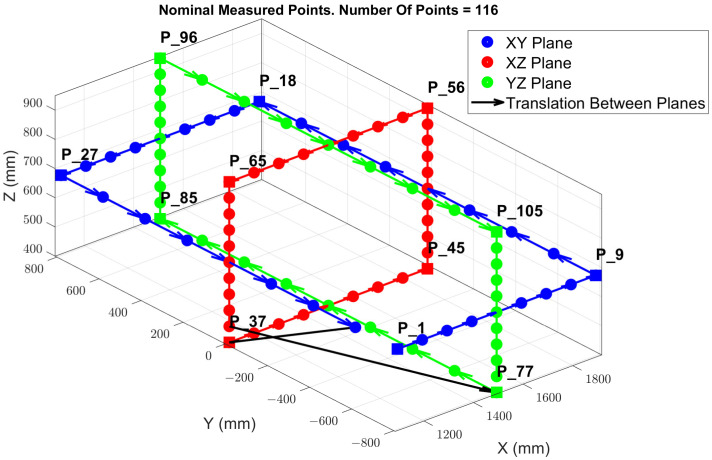
MIIM test point cloud configuration for 116 measurement points.

Figure 6 illustrates the arrangement of the point cloud configuration and the TCP locations, which are evenly distributed across the XY, XZ, and YZ planes, strategically positioned within the mid-range of each axis. This specific configuration of points is designed to streamline the process of inverse kinematics (IK), facilitating the resolution of the MT kinematic parameters that enable a potential volumetric correction. During the measurement phase, the table remains stationary, with no rotational movement; it undergoes only translational movements along the X, Y, and Z axes. Starting from point P1, the machine sequentially progresses to the subsequent point until it reaches the last point, completing a full cycle of measurements. Following this, the machine returns to point P1 to commence a new set of measurements.

The application of the MIIM method for the thermal characterisation of MTs necessitates the complete automation of the measurement process and establishes bidirectional communication with the MT controller to continuously map thermal errors. The primary challenge in automating the MIIM measurement process lies in orchestrating the pointing sequence from the laser tracker, which moves in tandem with the MT head, to each fiducial point,. To establish a spatial relationship among the integrated measurement instrument, fiducial points, and MT coordinate system, a spatial referencing process is executed through two best-fit alignment procedures. Initially, the relationship between the laser tracker’s coordinate system and the fiducial points is ascertained within a local coordinate system. Subsequently, this local coordinate system is aligned with the MT’s coordinate system [23].

Contrary to the traditional approach, the MIIM measurement strategy obviates the need for tracking capacity from the measurement instrument’s perspective [21]. This implies that the measurement technology must facilitate capturing and interrupting the beam between fiducial points to reset the distance datum within the measurement instrument for each fiducial point measurement. This capability is achieved through the utilization of Absolute Distance Measurement (ADM) technology. Additionally, the MIIM approach mandates a wireless instrument for either power or data transfer to the laptop and MT controller, especially during long-term MT thermal variation tests.

On the software front, the automation and management of the entire measurement process are facilitated using Spatial Analyzer (SA™) software (https://www.kinematics.com/spatialanalyzer/, accessed on 22 February 2024). The Unified Spatial Metrology Network (USMN) algorithm is employed instead of a pure multilateration approach [21]. This choice enables the development of tailored measurement solutions, exemplified in the case of the MIIM technique. Customised software has been devised to enable continuous and fully autonomous MIIM measurements on the MT spanning several days or months. This strategic integration of advanced hardware and software components underscores the comprehensive and innovative nature of the MIIM method in addressing the intricate challenges posed by thermal characterisation in the realm of machine tools.

### 2.4. The Uncertainty Budget of the Proposed Measurement Method

The uncertainty budget approach, as recommended by the “General Guide for the Evaluation of Measurement Data” (GUM) [26], provides a comprehensive framework for quantifying different sources of uncertainty and delineating their impact on the overall measurement outcome. Specifically, the ISO/TR 230-9:2005 [27] guideline provides information on estimating measurement uncertainty for procedures aligned with the ISO 230 series, highlighting the method’s effectiveness in addressing uncertainty factors.

Within the context of the proposed measurement method, significant sources of uncertainty include (a) uncertainty due to variations in environmental temperature (*u_ETVE_*), (b) uncertainty inherent in the measurement methodology (*u_MIIM_*), and (c) uncertainty stemming from compensation for the interferometer’s air refractive index (u_T_). Among these, measurement method uncertainty (*u_MIIM_*) is classified as a Type A contributor, whereas uncertainties related to environmental temperature variation error (*u_ETVE_*) and air refractive index compensation (*u_T_*) are categorised as Type B contributors [26].

In accordance with the GUM guidelines, Type A uncertainty is derived from the statistical analysis of a series of observations, while Type B is estimated from any non-statistical sources such as expert judgment, calibration certificates, or a manufacturer’s specifications. Thus, the combined standard uncertainty of the proposed measurement method is determined through the law of uncertainty propagation. For straightforward scenarios in which input parameters are uncorrelated and the underlying model is linear, the output quantity’s standard uncertainty is calculated using the root-sum-squared method, as detailed in Equation (1).

To ascertain the expanded measurement uncertainty (U) with a 95% confidence level (employing a coverage factor of *k* = 2), the calculation follows the formula presented in Equation (2).

(1) $$u_{TC}=\sqrt{u_{ETVE}^{2}+u_{MIIM}^{2}+u_{T}^{2}}$$

(2) $$U_{TC}=k\cdot u_{TC}$$

wherein the following definitions apply:

- U*_TC_*: The expanded measurement uncertainty of the proposed measurement method.
- *k*: The coverage factor, set at *k* = 2, corresponds to a confidence level of approximately 95%.
- *u_TC_*: The combined standard measurement uncertainty of the proposed measurement method.
- *u_ETVE_*: The standard measurement uncertainty contribution due to environmental temperature variation errors (ETVEs) effects on the MT.
- *u_MIIM_*: The standard measurement uncertainty contribution of the MIIM measurement method.
- *u_T_*: The standard measurement uncertainty contribution associated with compensating for the refractive index of air.

Table 3 presents a comprehensive uncertainty budget for the MIIM method. This table outlines each component of uncertainty associated with the MIIM technique, providing a detailed breakdown and analysis of the factors contributing to the overall measurement uncertainty.

The calculation of standard measurement uncertainty attributable to environmental temperature variation effects (ETVEs) on the machine tool, denoted as (*u_ETVE_*), follows the methodology outlined in Equation (3), as cited in Reference [9]. This process considers a rectangular distribution for the error, aligning with the assumption of a uniform likelihood of error values within a specified range. Consequently, this uncertainty component is classified as a Type B contribution, adhering to the classification criteria provided in Reference [27].

(3) $$u_{ETVE}=\frac{E_{ETVE}^{+}-E_{ETVE}^{-}}{2\sqrt{3}}$$

The estimation of the standard measurement uncertainty for the method is conducted using a Monte-Carlo simulation, according to the guidelines defined in JCGM 101:2008 [28]. This standard provides comprehensive instructions for utilising Monte-Carlo simulations to accurately estimate measurement uncertainties. Specifically, the simulation employs a mathematical model of the MIIM method, thoroughly incorporating all relevant uncertainty factors associated with MIIM. These factors include but are not limited to the distribution and number of fiducial points, the dimensions of the MT, and the error model of the measurement instrument. To ensure a robust analysis, the simulation is executed through 1000 iterations within the SA™ software, meticulously calculating the uncertainty contributions attributable to the MIIM measurement approach. 

In this research article, the standard measurement uncertainty contribution (*u_T_*) associated with compensating for the refractive index of air is determined using the Edlen formula, as referenced by Yan et al. [29]. To accurately quantify the uncertainty stemming from the application of the Edlen formula, the law of the propagation of variances is employed. The Edlen formula involves an in-depth analysis of the uncertainties associated with each input parameter—namely temperature, pressure, and humidity—and their cumulative effect on the uncertainty of the refractive index, as calculated using the formula. Sources for these uncertainties include calibration certificates, the specifications of the instruments, or the statistical analysis of data from repeated measurements. In scenarios in which the environmental condition sensors integrated into the laser tracker are deemed standard, an estimated standard uncertainty contribution of 0.8 micrometres per meter (µm/m) is derived. This estimate takes into account the precision of the sensors and their role in the overall uncertainty related to air refractive index compensation.

The results of the uncertainty measurements for each experimental test are detailed in Section 3 of this research article. These results were derived following the methodologies and procedures outlined in Section 2.4. 

## 3. Experimental Tests and Results

This research seeks to validate the MIIM method for quantifying the impact of ambient temperature variations on large-scale MTs. An extensive experimental investigation was conducted using a ZAYER ARION G™ MT across a variety of ambient temperature conditions from May 2021 to September 2022.

The research was carried out under the natural environmental conditions of a workshop, deliberately avoiding the use of costly heating or cooling solutions to simulate the expansive space where the MT was located. Selection of test days was strategically based on weather forecasts, facilitating the examination under diverse thermal environments. This strategy aimed to enhance the method’s applicability to practical industrial environments.

Throughout the experimental phase, two primary environmental conditions were identified:

- Quasi-stationary environment: Characterised by minor and slow temperature changes over 24 h, with fluctuations remaining below 1 °C.
- Cyclic fluctuation environment: Defined by more pronounced temperature changes over several days, with rates of change exceeding 0.5 °C per hour and total variations approaching 4.5 °C within a single day.

The evaluation of the MT was performed within a specified working volume of 800 mm (X-axis), 1600 mm (Y-axis), and 600 mm (Z-axis). The measurement volume was limited by a minimum required distance between the integrated laser tracker instrument and the retro-reflectors positioned on the measurement platform of 1.5 m. As a result, the MT’s measurement volume was represented through point clouds consisting of points ranging from 50 to 120 points distributed across the XY, YZ, and ZX planes and intersecting one another.

The results of the experimental tests revealed that ambient temperature is the primary source of thermal variability in the MT measurement setup. An in-depth examination further clarified the thermal distortion effects on the MT within both quasi-stationary and fluctuating thermal environments.

### 3.1. Environmental Thermal Dependency

The primary aim of this experimental research is to demonstrate that variations in ambient temperature are the predominant thermal influence on the thermal deformation observed in MTs. Specifically, this research aims to verify that thermal effects from other potential heat sources, such as heat induced by the mechanical movements of the MT during measurements, do not significantly affect the MT’s structural integrity.

To facilitate the implementation of the MIIM method, linear motions are required to position the laser tracker at every measurement point across the MT’s entire working volume. Although these movements can generate heat due to friction in motors, guides, bearings, and transmissions, careful programming of these movements at reduced speeds and accelerations helps mitigate such heat production. The MIIM method involves executing each movement slowly, pausing for about three seconds at each measurement point to perform the distance measurement, thereby minimising the introduction of thermal distortions from internal heat sources during the measurement acquisition process. Thus, the only heat source affecting the MT during these tests considered comprises ambient temperature changes.

For the MT’s rotary axes, preventive measures include disabling the cooling systems of the electrospindle or the rotary table hours before testing begins, effectively eliminating any additional heat sources. The rotary axes on the MT head and tool rotation are kept stationary, employing motor brakes or the spindle’s Hirth coupling system to lock the measurement positions and prevent additional heat generation from motor losses. 

To support the hypothesis that ambient temperature variations comprise the predominant thermal load during MIIM execution, the temperatures recorded during the experimental phase were rigorously analysed using two methods: (a) a graphical analysis involving a comparison of temperature signals and (b) a mathematical analysis using some well-known data analytics techniques. 

The graphical analysis indicates that the measured temperatures provide evidence of how all recorded temperatures, with a certain delay, mirror the ambient temperature. Variations in slopes and amplitudes are attributed to distinct heat transfer conditions and thermal inertia at each sensor location. It shows that temperatures at various MT structural components, like columns and the bridge, closely follow the ambient air temperature. Additionally, temperature readings from the ballscrews installed on the linear axes, despite the linear positioning movements required to reach each measurement point, show no significant increase, reinforcing the conclusion that ambient temperature variations comprise the sole thermal load affecting the MT during the MIIM process.

**Figure 7:**
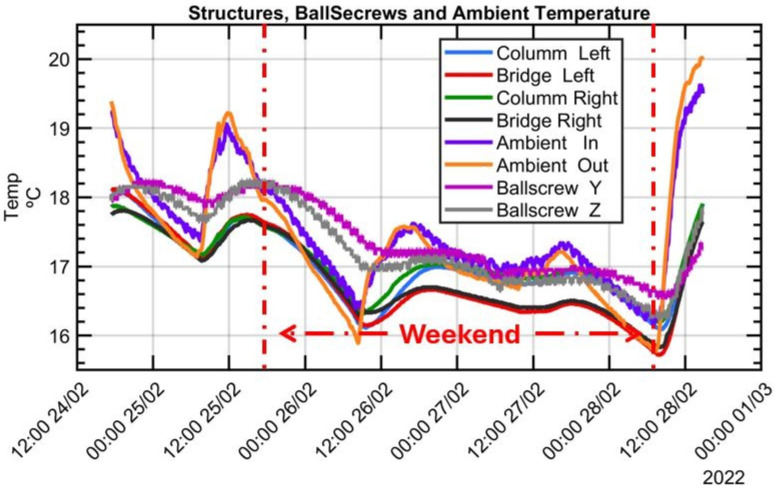
Evolution of temperatures on MT components during five-day test (weekend included).

Figure 7 showcases the temperatures recorded on the key components of the MT during the MIIM process carried out in February 2022. The ambient temperature exhibits noticeable daily fluctuations, showing a clear distinction between night and day cycles with similar patterns featuring lower maximum and minimum peaks, mirroring the ambient temperature evolution.

The second method utilises advanced mathematical techniques, making use of MATLAB’s statistical and machine learning toolboxes [30], to rigorously establish a correlation between temperatures measured on various components of the machine tool and fluctuations in environmental temperature.

In this research, the primary mathematical technique adopted is the principal component analysis (PCA), extensively detailed by Jollife et al. in their landmark publications [31,32]. As a refined statistical tool, the PCA boasts widespread applicability across a diverse array of disciplines. Its versatility is demonstrated in applications ranging from the thermal monitoring of MTs [33] and enhancing the efficiency of sensor networks [34] to the sophisticated analysis of thermal errors in complex mechatronic systems, frequently in conjunction with principal component regression (PCR) [11,35]. Consequently, the PCA method is considered one of the oldest and most widely used techniques for reducing the number of variables in large data sets while minimising information loss. It entails an orthogonal transformation of a set of original *p*-variables with redundant information into a smaller, optimal number of derived q-variables (principal components). The retained set of q variables captures variability, serving as a quality metric for the approximation or reduction of the original *p*-variables. A commonly accepted practice within the field is to retain principal components that account for at least 70% of the total variability, ensuring the dataset’s intrinsic structure is significantly preserved [32].

The primary objective of applying a PCA in this research study was to highlight the dominant influence of ambient temperature over all the temperature measurements taken on the MT components. To achieve this, three distinct PCAs were performed: a comprehensive analysis incorporating all collected signals and two focused analyses dedicated to internal and external sensors, respectively. The first principal component accounts for 87.5% of the variability in the data from external sensors and 69.7% for data from internal sensors, demonstrating that this component effectively summarises the information from both sensor sets.

The comparatively lower value captured by the first principal component for external sensors suggests an underlying influence; despite efforts to mitigate internal heat sources such as motors, ball screws, and bearings, there is still a certain level of influence on the recorded temperature. In this case, only one principal component just reaches the cut-off limit of accepted retained variability. These results strongly suggest that ambient temperature acts as the single source of variation, indicating that ambient temperature is the predominant factor influencing temperature measurement. This hypothesis is further corroborated when ambient temperature is excluded from the PCA analysis encompassing all signals, resulting in no principal component displaying a significant level of retained variability.

In conclusion, the PCA conducted in this study aimed to demonstrate the overriding influence of ambient temperature across all temperature measurements as well as confirm the correlation between recorded temperatures and the prevailing temperature. The results definitively position ambient temperature as the most important source of variability in temperature measurements. Additionally, it was found that the first principal component in the PCA effectively encapsulates the variability detected by both internal and external sensors, underscoring its utility in capturing the essence of temperature data. 

In contrast to the PCA approach, which simplifies data by generating a reduced set of dummy variables to represent a system, the Neighbourhood Component Analysis for regression (NCA) technique is employed to identify the variables that most effectively represent the system, simultaneously discarding those that do not contribute valuable information. The NCA distinguishes itself as an effective method for analysing relationships among a set of variables relative to a target variable and calculating their correlation indices. This capability makes the NCA particularly valuable in sensor networks for the purposes of sensor ranking and selection [36]. In the context of this study, an NCA was applied to explore the relationships between various temperature measurements and the ambient temperature, identifying those measurements that demonstrate the strongest correlation with the ambient temperature. This was achieved without the need to construct a theoretical model of the system, which is often a requirement of other methodologies.

**Figure 8:**
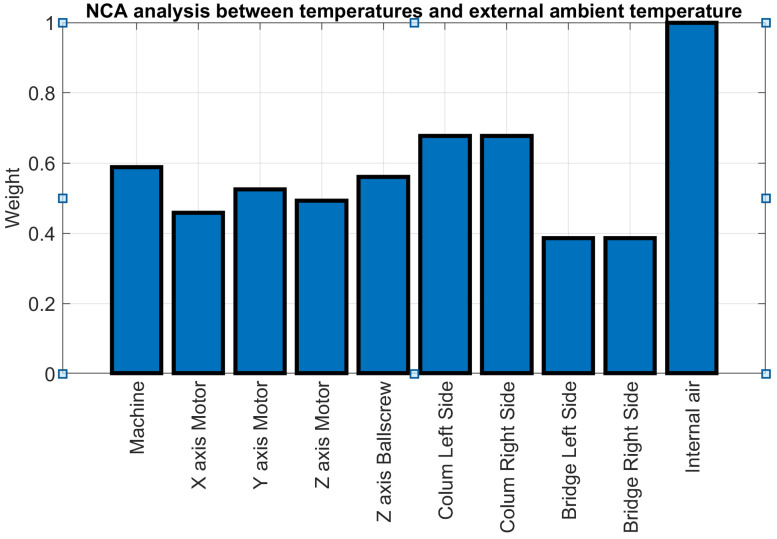
NCA analysis for temperature sensors compared to ambient temperature.

Figure 8 illustrates a selection of results from the NCA, highlighting the relationship between recorded temperatures and the external air temperature across the entirety of the data collected during the test campaign. The values have been normalised to unity, with the highest score designated as the benchmark. Notably, the internal air temperature attains the highest score, showing a close resemblance to the external air temperature. This similarity is attributed to the lack of thermal insulation within the enclosure, which is primarily intended to shield against fluids, chips, or any unexpected projectiles rather than provide thermal isolation.

The three analytical approaches (graphical analysis, PCA, and NCA) collectively affirm that the MIIM methodology does not introduce additional thermal variations to the MT, establishing environmental temperature variation as the sole source of thermal distortion. Furthermore, these analyses confirm a strong correlation between the temperatures measured on the primary MT components and the ambient temperature.

### 3.2. Quasi-Stationary Thermal Environment

During some of the test campaigns, the ambient temperature stability was remarkably high, providing quasi-stationary scenarios for evaluating the MT at different constant temperatures. This thermal setup mirrored the methodology of Gross et al. [1], albeit with a notable difference: in this study, a climatic chamber was not employed to stabilise the machine at predetermined temperatures for each test.

Over a period from May and July 2021, three sets of MIIM measurements were obtained on three different days, with each session extending over 14 h. For every MIIM set, comprehensive measurements of a 76-point cloud were conducted at intervals of 40 min, resulting in a total of 20 measurements per session.

**Figure 9:**
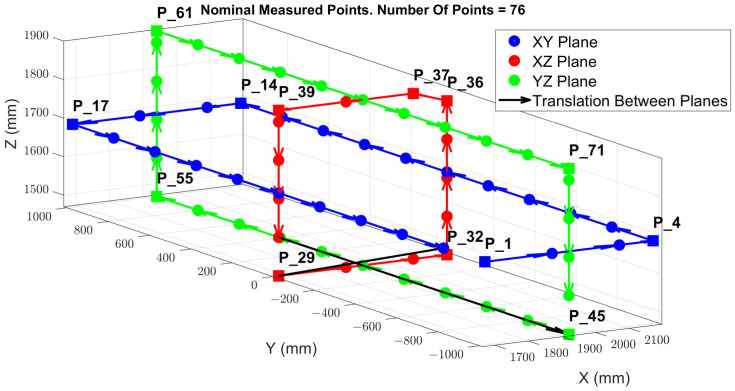
Measured points’ spatial distribution and movement sequence [23].

The MIIM methodology captures the coordinate data for each point, enabling an analysis of deviations in the X, Y, and Z axes relative to the nominal coordinates. Figure 9 showcases the spatial distribution of the 76 points measured, as well as the path followed to conduct the measurements across these three sessions.

During the duration of the tests, the ambient temperature exhibited minimal variation, staying within a tight range of less than 1 °C, resulting in a linear deformation of less than 10 µm/m on the MT’s linear axes. However, there was a notable absolute temperature disparity across the three MIIM sessions. In May, ambient temperatures ranged between 19 °C and 20 °C, but by the end of July, they had risen to around 24 °C. This temperature difference of 4.5 °C provided valuable insights into the MT’s geometry and underscored the MIIM methodology’s efficacy in examining environmental thermal effects on the MT.

**Figure 10:**
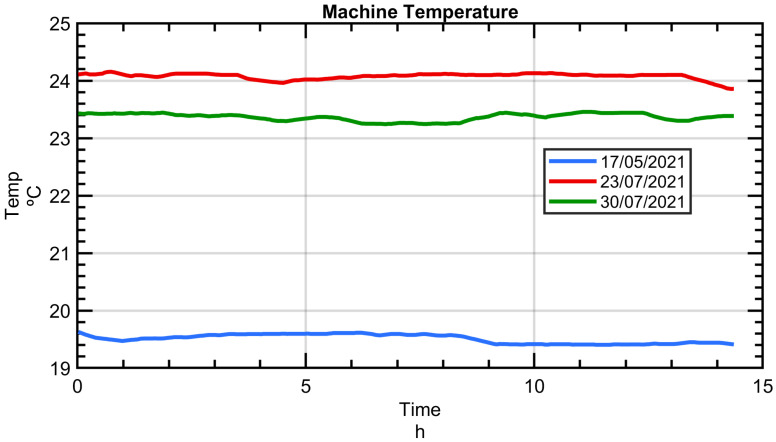
The temperature measured on the crossbeam during the MIIM tests performed between May and July of 2021 [23].

Figure 10 displays the temperature readings of the MT, specifically measured on the MT crossbeam (refer to Figure 3) across the MIIM tests conducted from May to July 2021. This temperature, referred to as “Machine”, serves as the MT reference temperature.

**Figure 11:**
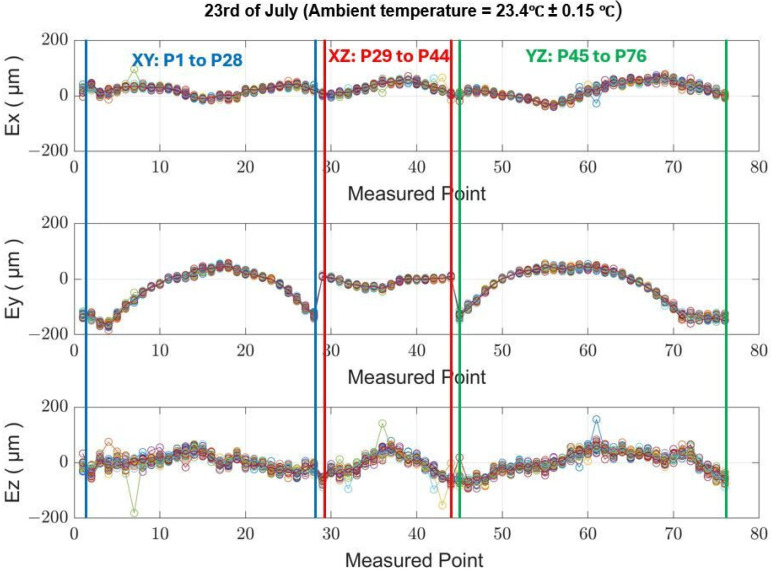
Measured point geometric deviation per MT axes Ex, Ey, and Ez (quasi-stationary MT condition).

Figure 11 illustrates the geometric deviation at each measured point across a series of 20 MIIM measurements conducted on 23 July 2021. The ambient temperature over the 14 h experiment fluctuated from a minimum of 23.2 °C to a maximum of 23.7 °C, resulting in an average value of 23.5 °C. The superimposed data within the figure, with a narrow band between them, underscore the minimal repeatability error of the MIIM methodology in contrast to the absolute geometric error of the MT. At the time of these tests, the maximum observed error was within ±0.1 mm. The geometric deviations from the targeted positions to the actual positions measured by MIIM at each point are denoted as Ex for the X axis, Ey for the Y axis, and Ez for the Z axis.

Table 4 shows the average standard deviation values for the X, Y, and Z axes, calculated for each day of measurement.

Analysing the results from the MIIM tests, it is observed that the standard deviation at each measurement point remains under 10 µm for the X and Y axes and under 15 µm for the Z axis. Given that the ambient temperature variation was maintained below 1 °C throughout the testing period, these standard deviation values primarily indicate the repeatability of the MIIM methodology. These data are crucial for the comprehensive characterisation of the MIIM approach. It is noteworthy that repeatability on the XY axes surpasses that of the Z axis, which may be attributed to poor system conditioning of the USMN algorithm. This is likely due to the positioning of a unique fiducial point outside the XY plane, affecting the algorithm’s conditioning and thereby influencing measurement accuracy differently across the axes. 

**Figure 12:**
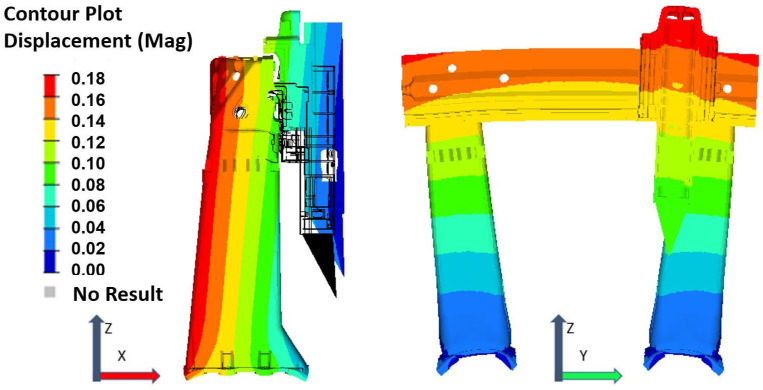
MT deformation (mm) at 23 °C (equilibrium temperature).

Figure 12 illustrates the theoretical thermal deformation of the MT in terms of shape and magnitude at an equilibrium temperature of 23 °C. These results were obtained from simulations using FEM and a steady-state analysis, offering a deeper understanding of the MT’s response under various thermal equilibrium states. The symmetrical design of the MT results in good behaviour at different stable temperatures, leading to almost linear MT structural deformation with ambient temperature variation. The methodology behind these experiments and comprehensive results are elaborated further in [23].

An FEM simulation of the MT provides detailed observations on its structural behaviour across varying ambient temperatures, specifically examining the XYZ orthogonal axes.

- X-direction: The MT’s TCP tends to move away from the crossbeam under a positive thermal gradient, indicating expansion, while it moves closer under a negative thermal gradient, indicating contraction. These movements are predominantly independent of influences from the MT’s Y and Z axes.
- Y-direction: Thanks to its symmetrical design, the MT maintains stability at the crossbeam’s centre, avoiding deformation. However, deformation escalates towards the ends of the Y-axis, particularly where the crossbeam connects with the supporting columns. The deformation along the Y-axis is primarily determined by crossbeam elongation/contraction and is slightly reduced when the ram extends.
- Z-direction: Both the columns and the ram exhibit linear deformations that directly correlate with ambient temperature changes. Such deformation increases when the ram is extended due to additional free length for elongation (positive thermal gradients) or contraction (negative thermal gradients). The MT table also experiences linear deformation, altering the distance between the workpiece (positioned on the table) and the TCP in response to ambient temperature shifts; this distance decreases as the temperature increases, and conversely, it increases as the temperature decreases.

**Figure 13:**
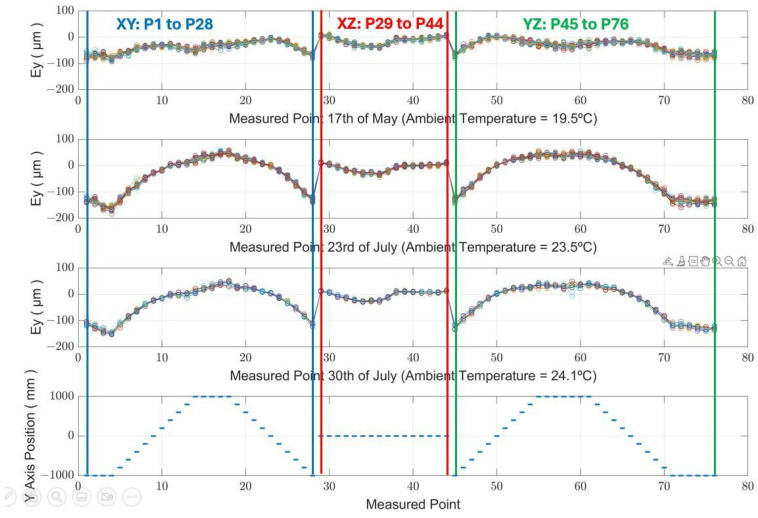
Deviation in the Y-direction. Ey obtained by the MIIM test, performed between May and July 2021.

Upon a closer examination of the Y-direction, which is the most affected axis, Figure 13 illustrates the correlation between the observed deviations along the Y-axis and the predictions made by the FEM, noting an increase in deviation values in tandem with rising ambient temperatures. The deviation, as it was calculated, is nearly negligible at the centre of the MT and symmetrically escalates towards the extremities of its travel range. 

The evaluation of measurement uncertainty adopts the uncertainty budget methodology, as detailed in Section 2.4. Within this framework, a central point from the measured point cloud obtained during the MIIM measurements is selected for an in-depth uncertainty budget analysis. The contribution to uncertainty (*u_ETVE_*) is determined by considering a rectangular distribution of the MT’s deviation at this central point, which is caused by variations in ambient temperature. The uncertainty contribution from the MIIM process (*u_MIIM_*) is derived from a Monte Carlo simulation utilising SA™ software. Furthermore, the uncertainty associated with temperature compensation (*u_T_*) incorporates the previously mentioned estimate, 0.8 µm/m. This estimate is applied to the MT’s operational volume under examination (X 800 mm; Y 1.600 mm; Z 600 mm) to calculate *u_T_*. Subsequently, the expanded measurement uncertainty is computed using Equations (1) and (2). Table 5 presents a detailed uncertainty budget for the quasi-stationary MT thermal experimental test, providing a comprehensive overview of the different sources of uncertainty and their impact on the measurement outcomes. In this scenario, the MIIM method emerges as the primary contributor to the total measurement uncertainty, which was expected due to minimal ambient temperature variations during the measurements, with a consistently similar contribution across all directions.

In summary, the quasi-stationary thermal conditions of the MT have been a valuable situation for accurately characterising the measurement uncertainty associated with the MIIM method and understanding the thermal errors of an MT at different temperatures. The experimental results demonstrate a strong correlation with the projected MIIM measurement uncertainty through simulations. Moreover, the observed deviations along the most affected direction (Y-axis) closely match predictions from FEM simulations regarding MT thermal distortion. This alignment underscores the efficacy of the MIIM method as a reliable approach for evaluating MT performance in stable-temperature environments, thus enabling the formulation and execution of efficient thermal compensation techniques.

### 3.3. Changing Thermal Environment

While prior experimental research was conducted under steady thermal conditions for the MT, real-world scenarios often present fluctuating ambient temperatures. These variations manifest as non-steady conditions, incorporating both diurnal and nocturnal thermal cycles over several days and distinct seasonal thermal fluctuations throughout the year.

As noted earlier, the workshop setting for this study was not subjected to artificial climate control, thereby situating the experimental research within in an uncontrolled thermal environment characterised by a fluctuating amplitude and frequency of thermal cycles. This decision was deliberate and aimed to capture the essence of real-world conditions. To this end, experimental tests were strategically scheduled during intervals forecasted to exhibit substantial daily temperature variations over several days. This carefully chosen timing guaranteed that the measurements accurately mirror actual operating conditions, thus significantly boosting the practicality and pertinence of the novel MIIM method under investigation for real-world MT applications.

**Figure 14:**
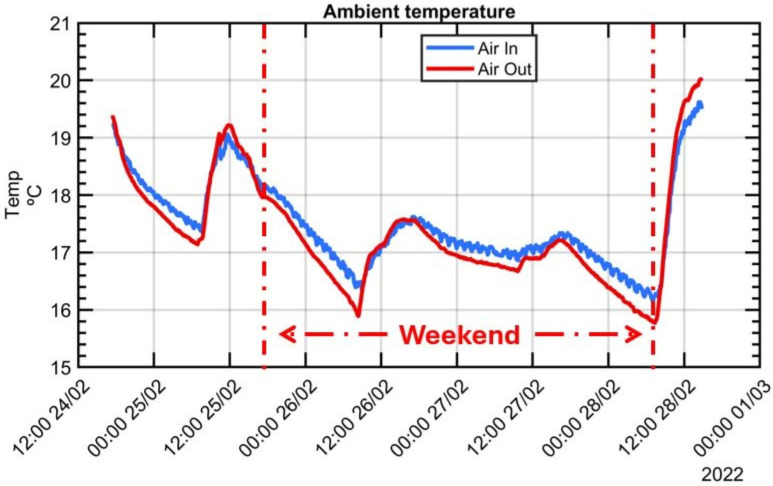
Ambient temperature variation during experimental tests performed between 24 February 2022 and 28 February 2022.

An experimental test conducted between 24 and 28 February, 2022 was performed in a changing thermal environment, with notable ambient temperature fluctuations recorded as follows: (a) an oscillation exceeding 2 °C was noted on the first day, reflecting the day cycle; (b) a gradual reduction in ambient temperature by more than 3.3 °C was observed from the morning of Friday, 25 February, to Saturday, 26 February, with a cooling rate of −0.16 °C/h over the weekend and was attributed to the deactivation of the shop floor’s heating system; (c) a period of stable ambient temperature prevailed throughout the weekend, and (d) a fast increment of 4.25 °C was registered on Monday, 28 February, with a temperature gradient of +0.61 °C/h in contrast to the workshop’s average environmental temperature change rate of approximately 0.5 °C/h. This sudden rise in ambient temperature was attributed to the reactivation of the shop floor’s heating system at 6 am. After it the test had to be terminated due to the necessity of utilising the machine for other purposes. Figure 14 illustrates the progression of air temperature evolution during the experiment, measured both outside and inside the working volume.

**Figure 15:**
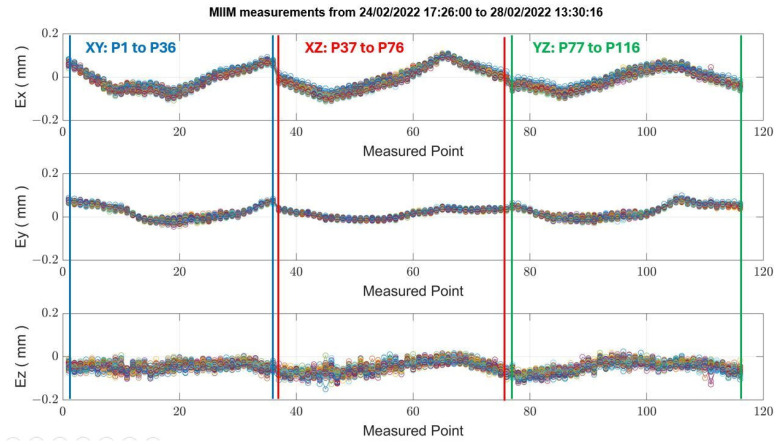
Deviation of measured points across MT axes (changing MT thermal condition).

Throughout the duration of the experimental test, the MIIM test was performed autonomously. For this experiment, a measurement point cloud of 116 points was established to enhance the resolution and facilitate the subsequent interpretation of MT thermal distortion, despite an extended measurement time of approximately one hour. Figure 15 illustrates deviations at each of the 116 measured points (refer to Figure 6) across 73 MIIM measurements conducted in February 2022, revealing that the peak absolute geometric error for the MT was ±0.16 mm at the time of the test campaign. Geometric deviations along the X, Y, and Z axes are represented as Ex, Ey, and Ez, respectively. Table 6 summarises the average and maximum standard deviation values recorded for the X, Y, and Z axes during the MIIM test campaign in February. The variability in ambient temperature likely contributed to the less optimal values compared to those obtained under more stable MT conditions. The fluctuations in ambient temperature are likely responsible for the less favourable results compared to those achieved under the quasi-stationary MT condition.

**Figure 16:**
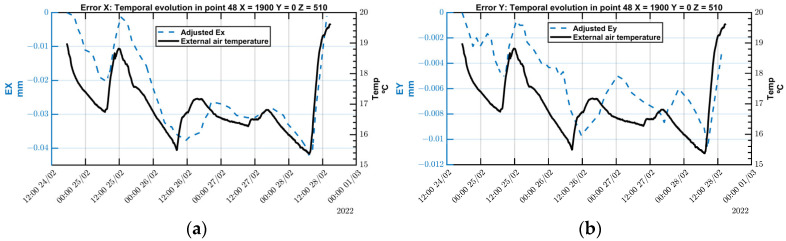
(**a**) Air temperature and Ex_ETVE_; (**b**) air temperature and Ey_ETVE_.

A key benefit of the MIIM method lies in its ability to track changes in measured point deviations over time. Figure 16 demonstrates the evolution of ETVE error in both X and Y directions at a specific point within the measurement point cloud. There is a clear correlation between temporal fluctuations in point deviation and changes in ambient temperature. Thus, given that ambient temperature is the sole source of thermal variations, the ETVE error accurately reflects the deviation at each measured point.

Regarding the deviation along the X-axis, Ex demonstrates a similar pattern to that of temperature fluctuations, albeit with a noticeable delay in its response to temperature changes. Specifically, as the temperature decreases, the error along the X-axis tends to increase in the negative direction. Conversely, an increase in temperature causes the error to shift in the opposite direction.

Similarly, the error along the Y-axis, Ey, follows a pattern that corresponds to ambient temperature changes. Despite theoretical predictions suggesting no variation error due to the MT’s symmetry, EY displays a variation of less than −10 µm in response to a total temperature decrease of −3.5 °C. This deviation, which is four times smaller than that observed in the X-axis, indicates that the MT does not deform symmetrically. This lack of symmetry may be attributed to the Y-slide nut’s location, which differs from that of the linear encoder measuring head.

In this context, the contribution of the air refractive index compensation (*u_T_*) becomes relatively insignificant, especially when compared to the environmental temperature variation error (*u_ETVE_*) contributor. Table 7 presents the expanded measurement uncertainty (U*_TC_*) for the MIIM method, calculated at a central point during the test campaign conducted on 24 February 2022.

In this instance, the MIIM method exhibits a contribution similar to that in the experiments conducted under stationary ambient temperatures (refer to Table 5), albeit slightly higher, likely due to the increased number of measured points necessitating more time for each measurement. For the X and Z axes, temperature emerges as the primary contributor as expected given the ambient temperature variations observed over the continuous five-day measurement period and the deformations along these axes, which are not fully symmetrical. Conversely, in the case of the Y axis, the lower contribution of temperature to the total measurement uncertainty is explained by the by the symmetrical expansion and contraction of the crossbeam that dominates the thermal deformation in the Y direction.

In conclusion, the findings confirm that the MIIM method has a minimal effect on thermal conditions during measurement activities. The expanded measurement uncertainty, derived from experiments conducted under varying MT thermal conditions, reveals a notable impact of the environmental temperature variation error (*u_ETVE_*) under the conditions of a fluctuating ambient temperature. Furthermore, the data on point deviations in these scenarios reveal a strong correlation between temperature changes and the deviations recorded at the measured points.

## 4. Conclusions

This research has rigorously evaluated Machine Integrated Inverse Multilateration (MIIM) as a pioneering approach for characterising thermal errors in large machine tools instigated by variations in environmental temperature. Acknowledging the critical importance of accurately evaluating large machine tools and the limited research dedicated to environmental thermal impacts, this research significantly broadens the scope of understanding thermal errors. While the prevailing research focus has been on errors originating from internal heating sources, such as drives, spindles, and the manufacturing process, this study highlights the substantial influence of ambient temperature variations. These variations become increasingly significant in larger machines, particularly those operated in large workshops with insufficient thermal regulation—a common scenario.

The manuscript demonstrates the deployment of the MIIM methodology on a Zayer Arion G™ large machine tool, and through extensive experimentation in environments with varying thermal conditions, it aims to refine MIIM and assess its effectiveness in identifying environmental thermal errors beyond the scope of a single machine’s performance evaluation. Conducted in a typical large workshop setting with minimal temperature control focused more on operator comfort than on precision thermal management, this research underlines the challenges of maintaining machine tool accuracy in environments lacking dedicated strategies for thermal regulation, thus providing comprehensive insight into the impact of ambient temperature fluctuations on large machine tool precision across diverse settings.

The experiments, conducted within the same workshop using the same machine, revealed two distinct thermal scenarios: quasi-stationary and dynamic. In the quasi-stationary environments, minor temperature fluctuations were observed between experiments, resulting in negligible thermal distortion during each test. On the other hand, dynamic thermal environments showcased marked day–night cycles and temperature variations over time, leading to more pronounced thermal errors in the machine.

Experiments carried out in quasi-stationary environments, defined by temperature variations of less than 1 °C over 14 h (or less than 0.07 °C/h), led to three key findings:

- The repeatability of the method was confirmed to be superior, with an accuracy better than 10 µm in the X and Y axes and within 15 µm in the Z axis under stable temperature conditions. This precision is in tight correlation with the uncertainty specifications of the AT960 Leica laser tracker used in the study.
- The experimental procedure was found to be non-contributory to any additional heat generation that could negatively impact the machine’s repeatability.
- The ability to conduct measurements under various steady-state temperature conditions facilitated the generation of distinct volumetric error compensation tables, enhancing the methodology’s application in precision engineering. In fact, the authors are currently addressing this issue. At an experimental level, multi-axis compensation at different temperatures has been successfully implemented using the functionalities provided by the Siemens 840 D control system. However, this research is still in a preliminary stage, and it was decided not to include it in this article as further testing is needed to draw significant conclusions.

During the realization of the experimental test, the temperature data collected were meticulously analysed through advanced statistical and machine learning techniques, such as the principal component analysis and Neural Connectivity Analysis. These analyses revealed that thermal variations and the resultant errors are solely attributable to fluctuations in environmental temperature. This insight validates the implementation strategy of the MIIM methodology, confirming that it does not introduce any significant thermal variations, thereby ensuring its accuracy and efficacy in isolating environmental impacts on thermal errors.

In conclusion, this research underscores the MIIM methodology’s effectiveness in evaluating the performance of large machine tools across different thermal environments. Through extensive testing, the automation process was validated as robust, enabling continuous, autonomous measurements without the need for manual intervention, even over several consecutive days. The results demonstrate that the MIIM methodology is a reliable solution for identifying thermal errors in large machine tools, delivering comprehensive data that meet the challenges outlined by Mayr et al. [2] for accurately assessing thermal errors. Additionally, the methodology’s potential for generalization to various machines and settings positions it as a valuable tool for characterising thermal errors in MTs driven by ambient temperature variations, highlighting its importance for improving machine tool precision and reliability.

The ability to perform volumetric error characterisation for medium- to large-sized machine tools every 40–60 min significantly expands the range of applications for the method. These include the fast validation of machines during factory acceptance tests as part of quality control processes, detailed characterisation of new machine models, and determining precision thresholds at final destinations in environments without thermal regulation. Future efforts, which have already begun, will extend the application of the methodology to larger machines and diverse configurations, targeting the analysis of broader working volumes. This expansion seeks to overcome the current limitations set by the minimum measurement distance of 1.5 m required between the laser tracker and retro-reflectors on the measurement table within this research. Moreover, the proposed MIIM strategy aims to identify key parameters critical for understanding the thermal dynamics of shop floors, such as ambient air velocity, the frequency and amplitude of temperature changes, the average ambient temperature, and temperature gradients, both horizontally and vertically, within the environment. These parameters are in line with those specified in ISO 230-3:2020 [9], indicating the methodology’s alignment with recognised standards and its potential to significantly enhance thermal error characterisation in manufacturing settings. 

The authors believe, and Zayer MT builder, as a direct collaborator on this research, confirms, that for the broader adoption of the MIIM method in the industry, the development of a new, cost-effective CNC instrument for distance measurement with enhanced accuracy is crucial. Such an innovation would significantly augment the effectiveness of the MIIM methodology in characterising thermal errors in large machine tools.

## Figures and Tables

**Table 1:** Main characteristics of additional thermal sensors.

Sensor Type	Measuring Range	Accuracy	Resolution	Reaction Time	Calibration
Pt-100	−100 to +260 °C	±(0.3 °C + 0.3% of mv)	0.01 °C	<45 s	ISO/IEC-17025 [24]

**Table 2:** Laser tracker and retro-reflector characteristics.

	Manufacturer	Model	Range	Angle	U (*k* = 2)
Laser Tracker	Hexagon (Nasdaq Stockholm, Sweden)	AT960	20 m		10 μm + 0.4 μm/m
Retro-reflectors	Hexagon	Super Cat-Eye	18 m	±75°	*

* Data not available; implicit in the results.

**Table 3:** Uncertainty budget for proposed measurement method.

Component of Standard Uncertainty (u)	Uncertainty Contributor	Contribution Type	Probability Distribution	Sensitivity Coefficient (c)
*u_ETVE_*	Error due to Environmental Temperature Variation	Type B	Rectangular	1.0
*u_MIIM_*	Uncertainty Inherent in the Measurement Method	Type A	Normal	1.0
*u_T_*	Uncertainty from Refractive Index of Air Compensation	Type B	Normal	1.0

**Table 4:** Average standard deviation values of MIIM test performed between May and July of 2021 (results in µm) [23].

	17 May 2021	23 July 2021	30 July 2021
X-axis	8.1	5.3	4.5
Y-axis	5.2	4.7	4.3
Z-axis	11.8	12.5	11.6

**Table 5:** The uncertainty contributors and expanded measurement uncertainty (U*_TC_*) of the MIIM method calculated at a central point for the test campaign performed on 23 July 2021.

	*u_ETVE_* (µm)	*u_MIIM_* (µm)	*u_T_* (µm)	U*_TC_* (µm)
X-axis	1.21	4.65	0.64	9.69
Y-axis	1.62	4.85	1.28	10.54
Z-axis	2.89	4.26	0.48	10.34

**Table 6:** Maximum and average standard deviation values for the MIIM test performed between 24 and 28 February (2022) (results in µm).

	X-Axis (μm)	Y-Axis (μm)	Z-Axis (μm)
Maximum	15	11	24
Average	11	5.3	14

**Table 7:** Expanded measurement uncertainty (U*_TC_*) of the MIIM method, calculated on a central point for the test campaign performed on 24 February 2022.

	*u_ETVE_* (µm)	*u_MIIM_* (µm)	*u_T_* (µm)	U*_TC_* (µm)
X-axis	13.86	5.50	0.64	29.85
Y-axis	3.46	5.15	1.28	12.67
Z-axis	10.97	5.67	0.48	24.71

## Data Availability

The data presented in this study are available on request from the corresponding author due to restrictions imposed by Zayer to perform this research using their machine.
